# Differences in Weight Status and Energy-Balance Related Behaviors among Schoolchildren across Europe: The ENERGY-Project

**DOI:** 10.1371/journal.pone.0034742

**Published:** 2012-04-25

**Authors:** Johannes Brug, Maartje M. van Stralen, Saskia J. te Velde, Mai J. M. Chinapaw, Ilse De Bourdeaudhuij, Nanna Lien, Elling Bere, Victoria Maskini, Amika S. Singh, Lea Maes, Luis Moreno, Nataša Jan, Eva Kovacs, Tim Lobstein, Yannis Manios

**Affiliations:** 1 Department of Epidemiology and Biostatistics and EMGO Institute for Health and Care Research, VU University Medical Center, Amsterdam, the Netherlands; 2 Department of Public and Occupational Health and EMGO Institute for Health and Care Research, VU University Medical Center, Amsterdam, the Netherlands; 3 Department of Movement and Sport Sciences, Ghent University, Ghent, Belgium; 4 Department of Nutrition, University of Oslo, Norway; 5 Department of Public Health, Sport and Nutrition, University of Agder, Kristiansand, Norway; 6 Department of Nutrition and Dietetics, Harokopio University, Athens, Greece; 7 Department of Public Health, Ghent University, Ghent, Belgium; 8 GENUD (Growth, Exercise, Nutrition and Development) Research Group. E.U. Ciencias de la Salud, Universidad de Zaragoza, 50009 Zaragoza, Spain; 9 Slovenian Heart Foundation, Ljubljana, Slovenia; 10 Department of Paediatrics, Pecs University, Pecs, Hungary; 11 IASO, International Association for the Study of Obesity, London, United Kingdom; University of Sao Paulo, Brazil

## Abstract

**Background:**

Current data on the prevalence of overweight and energy-balance behaviors among European children is necessary to inform overweight prevention interventions.

**Methodology/Principal Findings:**

A school-based survey among 10–12 year old children was conducted in seven European countries using a standardized protocol. Weight, height, and waist circumference were measured; Engagement in physical activity, sedentary and dietary behaviors, and sleep duration were self-reported. Descriptive analyses were conducted, looking at differences according to country, gender, and parental education. 7234 children (52%girls; 11.6±0.7 years) participated. 25.8% and 5.4% of boys, and 21.8% and 4.1% of girls were overweight (including obese) and obese (according to International Obesity Task Force criteria), respectively. Higher prevalence of overweight/obesity was observed in Greece, Hungary, Slovenia and Spain than in Belgium, Netherlands and Norway. Large differences between countries were found in intakes of sugar-sweetened beverages, breakfast, active transport, TV and computer time. More favorable overweight status and behavior patterns were found in girls than boys and in children of higher educated parents than in children of lower educated parents.

**Conclusions/Significance:**

High levels and striking differences in overweight status and potential risk behaviors were found among schoolchildren across Europe.

## Introduction

Overweight and obesity are important determinants of avoidable burden of disease [1], [2]. Overweight and obesity track from childhood into adulthood [3], [4]. Preventing overweight and obesity and promoting healthy energy-balance related behaviors (EBRB) during childhood are therefore important health policy priorities in Europe and beyond. To curb the obesity epidemic, up-to-date data on prevalence of overweight, obesity, associated EBRBs and groups at risk for overweight is essential. In earlier studies large differences in childhood overweight and obesity between European countries have been reported [5]. However, data of these overviews come from different studies, conducted in different years, are outdated for some countries [6], or relied on self-reported weight and height [7], [8].

Recent reviews suggest that high intakes of sugar sweetened beverages, breakfast skipping, lack of physical activity, high levels of ‘screen time’ (e.g. watching TV, or playing e-games), and lack of sleep [9], [10] are associated with overweight and obesity among school-aged children [11]–[14]. Earlier research indicate that the prevalence of overweight and EBRB differ between girls and boys and is considerably higher among youth from lower socio-economic backgrounds [7], [15]–[26]. Cross-European studies providing insight into engagement in EBRBs assessed by standard methodology, and their socio-demographic correlates are lacking. The present study presents data from the “EuropeaN Energy balance Research to prevent excessive weight Gain among Youth” (ENERGY)-project on measured weight status and reported EBRB among 10–12 year olds in and differences between seven European countries [27]. The specific research questions are:

- What are the distributions and differences in body mass index (BMI), waist circumference (WC), overweight and obesity in schoolchildren in seven countries across Europe?
- What are the distribution and differences in dietary, physical and sedentary activities and sleeping duration in these countries across Europe?
- What are differences in anthropometrics and EBRB according to sex and parental education across different countries in Europe?

## Methods

A description of the rationale and organization of the ENERGY-project [27] and a comprehensive description of the design, procedures, and methodology of the ENERGY school-based survey [28] are published elsewhere. The data collection manual and survey questionnaires for the Energy cross-sectional survey are available online http://projectenergy.eu. The project adhered to the Helsinki Declaration and the conventions of the Council of Europe on human rights and biomedicine. All participating countries obtained ethical clearance from the relevant ethical committees and ministries. in Belgium the survey was approved by the Medical Ethics Committee of the University Hospital.

Ghent; in Greece the survey was approved by the Bioethics Committee of Harokopio University; in Hungary the survey was approved by the Scientific and Ethics Committee of Health Sciences Council; in The Netherlands the survey was approved by the Medical Ethics Committee of the VU University medical center; in Norway the survey was approved by the National Committees for Research Ethics in Norway; in Slovenia the survey was approved by the National Medical Ethics Committee of the Republic of Slovenia; and in Spain the survey was approved by Clinical Research Ethics Committee of the Government of Aragón. Furthermore, research permission was, if necessary, obtained from local school authorities (local school boards and/or headmasters).

### Sampling and Respondents

The school-based survey was carried out between March and July 2010 in Belgium, Greece, Hungary, the Netherlands, Norway, Slovenia, and Spain, among pupils in the final years of primary education (aged 10–12 years). These seven countries were selected to provide variation across regions in Europe and thus variation in potential obesogenic behaviors and prevalence of overweight and obesity. Inclusion of more countries across Europe was not possible because of budgetary restraints. Based on previous cross-European studies (e.g. the Pro-Children study [29]) a minimum sample of 1,000 schoolchildren per country and one parent/caretaker for each child was aimed for. Equal numbers of participants from each country were included to allow between country comparisons. Sampling was national in Greece, Hungary, the Netherlands, and Slovenia. In Spain, schools in the region of Aragón were selected; Belgium selected schools from Flanders and Norway selected schools from the southern regions of the country [28]. Within each country or region, three provinces were randomly selected from each of the lowest, mid and highest tertiles of degree of urbanisation (i.e. the percentage of inhabitants living in towns of >20,000 persons). A municipality of >20,000 inhabitants from each selected province was randomly chosen, with schools randomly selected for inclusion in the study from all schools in that municipality. The clustering of the survey was taken into account in sample size calculations.

A school recruitment letter was sent to the headmaster or principal of the sampled schools, followed by a personal telephone call. Following the school’s agreement, parents received a letter explaining the study purpose and were asked for written consent for their child’s and own participation.

### Measures

Measurements were conducted according to standardized protocols. Dietary, physical activity and screen viewing behaviors were assessed by the child questionnaire. Child’s sleep duration was reported by the parent. The children completed questionnaires and anthropometric measurements during school time. Test-retest reliability was tested by administrating the questionnaire twice with a one week interval among 720 schoolchildren across the participating countries. The intraclass coefficients and percentage agreement was good to excellent for 77% of items and construct validity was moderate to excellent for 73% of the items [30]. In the paragraphs hereafter information on the Intraclass coefficients (ICC) is provided for the specific questionnaire items. Detailed information regarding the procedures, training of research staff, development of the questionnaire [28], and test- retest reliability and construct validity [30] is published elsewhere.

#### Weight, Height and waist-circumference (WC)

Body height, weight, and WC were measured by trained research assistants. The children were measured in light clothing without shoes. Body height was measured with Seca Leicester Portable stadiometer (accuracy of 0.1 cm). Weight was measured with a calibrated electronic scale SECA 861 (accuracy of 0.1 kg), WC with the SECA 201 measuring band (accuracy 0.1 cm). Two readings of each measurement were obtained. If the two readings differed more than 1%, a third measurement was taken. Body mass index (BMI) and overweight status (overweight, obesity) based on the International Obesity Task Force criteria (IOTF) [31] as well as the WHO criteria [32] were calculated to enable comparison with other studies that applied either of these criteria.

#### Dietary behaviors

Intakes of soft drinks and fruit juices were each assessed with two food frequency questions (FFQ). First children were asked on how many days per week they drank the beverage on a seven point scale ranging from never to more than once every day (ICC soft drink = 0.72; ICC fruit juice = 0.64). Subsequently they were asked to indicate how much they drank on days they consumed the beverage by ticking the number of glasses (or small bottles, i.e. 250 ml; ICC = 0.59), cans (i.e. 330 ml; ICC = 0.53)) and/or large bottles (i.e. 500 ml; ICC = 0.59) for soft drinks, or glasses/small cartons (250 ml; ICC = 0.54) and/or regular cartons (330 ml; ICC = 0.52) for fruit juices. Pictures of the serving sizes were printed in the questionnaire. Mean intake in ml per day was calculated from the FFQ by multiplication of number of days per week and amount per day in ml divided by 7. Additionally, children were asked to indicate how much of the beverages they had consumed on the day before questionnaire administration, again by ticking the number of glasses (ICC = 0.58), cans (ICC = 0.53) and/or bottles (ICC = 0.55) for soft drinks, and glasses (ICC = 0.48) and/or cartons (ICC = 0.35) for juice.

Breakfast habits were assessed by two questions asking the children on how many schooldays per week (ICC = 0.73) and on how many weekend days (ICC = 0.52) they normally had breakfast. Breakfast frequency per week was calculated by adding up the answers of the two questions. The frequency score was recoded into a skipping breakfast score ([0] had breakfast 7 days/week; [1] had breakfast 0–6 times/week).

#### Physical activity behaviors

Transport to school was assessed by two questions about how many days per week the child cycled (ICC = 0.94) or walked (ICC = 0.91) to school (from never [0] to 5 days/week [5]) and two questions on how long the bike ride (ICC = 0.81) or walk (ICC = 0.70) to school was (4 answering categories, from 1–5 minutes to more than 15 minutes). Total bike/walk time per week was calculated by multiplying the number of days with the mean time of the answering category times 2 (i.e. it was assumed that if a child cycled or walked to school, the same mode of transportation was taken on the way back). Total active transport was calculated by adding up total bike and walk times. Finally, a question was included on what mode of transportation was used to get to school on the day of administration (ICC = 0.79).

Regarding organized sports participation, questions were included on how many hours per week children participated in sports for two sports (ICC = 0.74 for sport 1; ICC = 1.00 for sport 2). Based on the answers average time of sport participation per week was calculated for each child.

#### Sedentary behaviors and sleeping

Screen time questions (i.e. TV and computer time) were asked about time spent watching TV (including video and DVD;) and computer (PC) activities for weekdays (ICCs = 0.67 for TV and for PC) and weekend days (ICCs = 0.68 for TV and 0.67 for PC) separately using a categorical scale ([0] not at all, [0.5] 30 minutes per day… [4] 4 hours per day). Mean TV, PC and total screen time per day were calculated. Finally, the children were asked to indicate how much they spent watching TV (ICC = 0.68) or used the computer (ICC = 0.54) the day before questionnaire administration.

The child’s sleep habits were reported by the parent about how many hours the child sleeps on average per night, separately for weekdays (ICC = 0.81) and weekend days (ICC = 0.78). A mean number of hours of sleep per day was calculated.

Parental education was also assessed in the Parent Questionnaire. Parents were asked to report their own level of education and that of the other parent/caregiver. Both scores were combined, and dichotomized into low (0, both parent/caregivers with fewer than 14 years of education) and high (at least one parent/caregiver with 14 or more years of education).

### Analysis

Multivariate Analyses of Variance (MANOVA) were performed to assess differences in means or proportions according to country, gender and parental education with anthropometrics (i.e. BMI, WC, % overweight/obese, according to IOTF as well as WHO criteria) and potential risk behaviors as dependent variables and country, sex and parental education as factors, and with age as a covariate. For the dependent variables that showed significant results, subsequent pairwise comparisons by means of ANOVAS with Bonferoni correction were conducted. For EBRBs means and standard deviations are reported, but because of skewed distributions, a table with median values is made available as an additional file (see Tables S1 and S2). Differences according to gender and parental education were tested in each country separately.

## Results

### Participant Characteristics

Between 15 (Slovenia) and 37 (Greece) schools participated, with a wide range in response rates at school level (see Table 1). Response rates at the child level were in general high (>80%), except for Hungary, Norway and Spain where lower response rates were obtained, mainly because of lack of parental consent. Almost all children who completed the questionnaire also participated in anthropometrics measurements. For the parent questionnaire high response rates were obtained except for Belgium and the Netherlands (see Table 1). The total sample comprised 7,234 children (Mean age 11.6±0.7 years, 52% girls).

**Table 1:** Overview of data collection and response rates per country.

	Belgium	Greece	Hungary	Netherlands	Norway	Slovenia	Spain
Schools recruited, N	26	37	29	23	21	15	24
% response	29%	54%	71%	5%	36%	100%	72%
Completed child questionnaires, N	1003	1077	1022	926	1004	1178	1024
% response^1^	82%	94%	100%	93%	99%	100%	100%
% response^2^	77%	64%	33%	92%	45%	98%	43%
Completed child anthropometrics, N	1005	1077	1022	898	980	1146	1012
% response	82%	94%	100%	86%	97%	97%	99%
Completed parent questionnaires, N	763	1008	932	404	903	1028	964
% response^3^	62%	83%	91%	41%	89%	87%	94%
% response^4^	59%	60%	67%	40%	41%	86%	40%

1 response rate is the percentage of children that completed the child questionnaire of the children with parental consent.

2 response rate is the percentage of children that completed to child questionnaire of the total number of eligible children.

3 response rate is the percentage of children with completed parent questionnaire of the children with parental consent.

4 response rate is the percentage of children with completed parent questionnaire of the total number of eligible children.

### Anthropometrics

Multivariate Analyses of Variance revealed a significant multivariate main effect on the antropometric variables for country (Willk’s λ = 0.88, F (36, 30878.0) = 25.07, p<0.00), gender (Willk’s λ = 0.93, F(6.0, 7036.00) = 91.31, p<0.00), and parental education (Willk’s λ = 0.98, F(6.0, 5082.00) = 14.85, p<0.00).

Across the countries, 25.8 and 5.4% of the boys, and 21.8 and 4.1% of the girls were overweight (including obesity) and obese, respectively (Table 2). Mean BMI varied between 18.3 kg/m^2^ (Belgium) and 20.6 kg/m^2^ (Greece) for boys and 18.1 kg/m^2^ (Belgium) and 20.3 kg/m^2^ (Greece) for girls. Highest WCs were observed in Greece – both in boys and girls; the lowest WCs were found in the Netherlands and Belgium. The difference in WC between Greece and the Netherlands was almost 8 cm for boys and 7 cm for girls. The highest prevalence of overweight (including obesity) was also observed in Greece, and the lowest in Belgium (Girls) and Norway (Boys). In Belgium, Norway, and the Netherlands prevalence rates were significantly lower than other countries. All countries had significantly lower prevalence of overweight/obesity than Greece. Similar patterns were found for obesity separately, but in Spain low obesity rates were observed despite a relatively high overweight prevalence. Significantly higher overweight prevalence was observed in boys than in girls in the whole sample. Children of high educated parents had a lower BMI and WC and were less likely to be overweight or obese than children of lower educated parents (Table 2, bottom half). These differences showed similar patterns across countries.

**Table 2:** Means, standard deviations (SD) for Body Mass Index (BMI) and waist circumference (WC) and percentage overweight and obese (using IOTF and WHO criteria) in each of the participating countries, for boys and girls separately and for children with low educated and high educated parents separately.

	Belgium		Greece		Hungary		Netherlands		Norway		Slovenia		Spain		Differences between countries^a^	
	(B)		(Gr)		(Hu)		(Nl)		(N)		(Sl)		(Es)			
Gender	Boys	Girls	Boys	Girls	Boys	Girls	Boys	Girls	Boys	Girls	Boys	Girls	Boys	Girls	Boys	Girls
	N = 481	N = 522	N = 503	N = 588	N = 458	N = 562	N = 453	N = 448	N = 470	N = 508	N = 559	N = 589	N = 485	N = 523		
BMI (mean, SD)^a^	18.3 (3.2)	18.1 (3.0)	20.6 (3.7)	20.3 (3.9)	19.7 (3.8)	19.3 (3.5)	18.4 (3.2)	18.5 (3.0)	18.4 (2.4)	18.7 (3.0)	19.4 (3.5)	18.9 (3.2) *	19.1 (2.8)	19.2 (3.0)	B,Nl,N<Gr,Hu,Sl,EsHu,Sl,Es<Gr	B<Gr,Hu,Sl,EsN,Nl<Gr,Sl,EsHu,Sl,Es<Gr
WC (mean, SD)^b^	64.1 (8.5)	62.6 (7.6)**	71.8 (10.2)	69.4 (9.2)***	70.1 (11.0)	66.9 (9.2) ***	64.0 (7.9)	62.6 (7.0)**	65.0 (6.7)	63.9 (7.3)*	68.9 (9.6)	65.9 (8.3)***	66.3 (7.4)	64.9 (7.2)**	B,N,Nl<Gr,Hu,Sl,EsHu,Sl<GrEs<Gr,Hu,Sl	B,N,Nl<Gr,Hu,Sl,EsHu,Sl,Es<Gr
% Overweight^c^	16.9	13.5	44.4	37.7*	27.7	22.6*	16.8	15.4	15.1	13.8	31.7	22.5**	25.8	23.8	B,N<Gr,Hu,Sl,EsHu,Sl,Es<GrNl<Gr,Hu,Sl	B,N<Gr,Hu,Sl,EsHu,Sl,Es<GrNl<Gr,Hu,Es
% Obese^c^	3.7	2.3	11.2	9.7	6.8	4.1*	4.5	2.5	0.4^##^	2.4	7.5	3.9*	2.9	3.1	B,Nl<GrN<Gr,Hu,SlEs<Gr,Sl	B,Hu,Nl,N,Sp,Es<Gr
%Overweight WHO^d^	24.5	17.9**	55.4	44.1***	35.5	26.2**	26.0	18.5**	21.2	17.8	39.5	28.3***	37.6	30.8*	B,Nl,N<Gr,Hu,Sl,EsHu,Sl,Es<Gr	B,N<Gr,Hu,Sl,EsNl<Gr,Sl,EsHu,Sl,Es<Gr
%Obese WHO^d^	8.0	4.1**	27.6	14.8***	14.0	6.8***	7.2	4.2	3.9	4.2	16.8	6.2***	10.7	6.1**	Es,Hu,Sl<GrB,Nl,N<Gr,Hu,Sl	B,Hu,Nl.N.Sl.Es<Gr

* P<0.05;

** p<0.01;

*** p<0.001 girls significantly lower than boys, adjusted for age;

‡ p<0.05 boys significantly lower than girls, adjusted for age;

^ p<0.05;

^̂ p<0.01;

^̂̂ P<0.001 high educated significantly lower than low educated, adjusted for age;

a pairwise comparison between countries using Bonferroni correction, adjusted for age;

b pairwise comparison between countries using Bonferroni correction, adjusted for age and height;

c overweight categories based on the IOTF criteria, including both overweight and obesity;

d Weight categories based on WHO criteria, overweight including obese stands for BMI+1SD; obese stands for BMI+2SD.

### Energy-balance Related Behaviors

MANOVA showed a significant multivariate main effect on the energy balance related behaviors for country (Willk’s λ = 0.242, F(102.00, 28716.63) = 79.626, p<0.00), gender (Willk’s λ = 0.921, F(17.00, 5041.00) = 25.276, p<0.00), and parental education (Willk’s λ = 0.937, F(17.0, 4456.00) = 17.59, p<0.00).

#### Dietary behaviors

Table 3 (Means and standard deviations) and Table S1 (Medians and quartiles), show large differences across countries in dietary behaviors as identified by the post-hoc analyses of variance. Based on the 24-h recall questions, soft drink intake ranged from more than 600 ml/day among Hungarian and Dutch boys to less than 150 ml/day in Greek and Spanish girls. In all countries except Hungary, boys had significantly higher intakes than girls. Fruit juice intake was high in Dutch boys, bringing their mean sugary drinks consumption to more than one liter/day. Gender differences in fruit juice consumption appeared to be smaller than for soft drinks. The FFQ assessments resulted in somewhat higher mean intake, but in similar patterns regarding country comparisons (lower 24-h recall results may be due to questionnaire assessments not being done on Mondays, therefore excluding weekend days from the 24-h recall assessment).

**Table 3:** Means and standard deviations (SD) for dietary behaviors in each of the participating countries, for boys and girls separately and for children with low educated and high educated parents separately.

	Belgium		Greece		Hungary		Netherlands		Norway		Slovenia		Spain		Differences between countries a	
	(B)		(Gr)		(Hu)		(Nl)		(N)		(Sl)		(Es)			
Gender	Boys N = 477	Girls N = 516	Boys N = 500	Girls N = 586	Boys N = 459	Girls N = 563	Boys N = 456	Girls N = 463	Boys N = 450	Girls N = 518	Boys N = 570	Girls N = 601	Boys N = 493	Girls N = 529	Boys	Girls
Soft drink FFQ (ml/day)	502±593	408±515*	139±216	92±186 ***	608±648	496±576 **	700±633	565±525 **	275±328	174±233 ***	413±557	249±432 ***	239±348	133±246 ***	B.Hu<NlGr<B,Hu,Nl,N,Sl,EsN,Es<B,Hu,Nl,SlSl<Hu,Nl	B,Hu<NlGr,N,Es<B,Hu,Nl,SlSl<B,Hu,Nl
Soft drink-24 h recall (ml/day)	381±466	309±390*	179±259	133±226 **	641±630	591±600	689±586	599±443*	240±379	168±264 **	498±604	354±447 ***	203±319	123±249 ***	B<Hu,Nl,SlGr,N,Es<B,Hu.Nl,SlSl<Hu,Nl	B,Sl<Hu,NlGr,N,Es<B,Hu,Nl,Sl
Fruit Juice FFQ (ml/day)	265±300	251±297	286±292	241±238 **	349±442	345±397	384±434	385±399	217±281	159±207 ***	415±v569	355±389 *	250±258	223±270	B,N,Es<Hu,Nl,SlGr<Nl,SL	B,Gr,Es<Hu,Nl,SlN<B,Gr,Hu,Nl,Sl,Es
Fruit juice-24 h recall (ml/day)	271±323	226±264*	254±300	224±232	305±416	331±420	388±413	391±390	175±284	132±209 **	413±468	370±382	220±278	207±234	B,Gr,Hu<Nl,SlN<B,Gr,Hu,Nl,SlEs<Hu,Nl,Sl	B,Gr,Es<Hu,Nl,SlHu<NlN<B,Gr,Hu,Nl,Sl,Es
Breakfast (days/week)	6.2±1.6	6.2±1.6	5.4±2.1	5.4±2.2	5.8±1.9	5.6±1.9	6.4±1.4	6.3±1.6	6.3±1.6	6.5±1.3	5.1±2.2	5.1±2.2	6.6±1.1	6.7±1.0	B<EsGr,Hu<B,Nl,N,EsSl<B,Hu,Nl,N,Es	B<N,EsGr,Hu<B,Nl,N,EsSl<B,Hu,Nl,N,Es
Skipped breakfast ≥ 1/week (%)	30.4	30.4	47.8	46.1	38.1‡‡	47.5	24.1	22.1	21.8	19.8	51.7	51.3	16.9	12.0*	B,Hu<Gr,SlNlN<Gr,Hu,SlEs<B,Gr,Hu,Sl	B<Gr,Hu,SlNl,N,Es<B,Gr,Hu,Sl
**Parental education**	**Low N = 105**	**High N = 561**	**Low N = 430**	**High N = 461**	**Low N = 320**	**High N = 443**	**Low N = 78**	**High N = 271**	**Low N = 183**	**High N = 533**	**Low N = 392**	**High N = 503**	**Low N = 171**	**High N = 708**	**Low**	**High**
Soft drink FFQ (ml/day)	565±559	350±473 ^̂̂	119±194	86±152 ^̂	654±679	469±565 ^̂̂	631±608	505±491	251±303	208±277	344±550	257±393 ^	256±411	163±269 ^̂̂	Gr<B,Hu,Nl,Sl,EsN,Sl,Es<B,Hu,Nl	B<Hu,NlGr<B,Hu,Nl,N,Sl,EsN,Sl<B,Hu,NlEs<B,Hu,Nl,Sl
Soft drink-24 h recall (ml/day)	472±465	279±358 ^̂̂	174±249	121±200 ^̂̂	633±595	564±604	638±476	559±460	233±291	167±315 ^̂	379±560	319±446 ^̂̂	216±377	143±254 ^̂	Gr,N,Es<B,Hu,Nl,SlSl<Hu	B,Sl<Hu,NlGr,N,Es<B,Hu,Nl,Sl
Fruit Juice FFQ (ml/day)	297±342	233±274	259±270	258±246	371±469	338±396	437±431	303±325 ^̂	165±213	199±257	405±446	336±385 ^	285±338	226±241 ^̂	Gr,N<Hu,Nl,SlEs<Sl	B,Es<Hu,Nl,SlGr<Hu,SlN<Gr,Hu,Nl,Sl
Fruit juice-24 h recall (ml/day)	264±334	229±269	240±246	219±234	330±408	300±405	455±394	294±304 ^̂̂	128±232	162±256	423±473	336±370 ^̂	264±336	200±222 ^̂	B,Es<Nl,SlGr<Hu,Nl,SlHu<SlN<Gr,Hu,Nl,Sl,Es	B<Hu,SlGr,Es<Hu,Nl,SlN<B,Hu,Nl,Sl
Breakfast (days/week)	5.8±1.9#	6.2±1.5	5.3±2.1#	5.7±2.0	5.7±1.9	5.6±2.0	6.5±1.2	6.6±1.1	6.3±1.5	6.5±1.3	5.0±2.2##	5.5±2.1	6.5±1.1	6.7±1.0	Gr<Hu,Nl,N,EsHu<Nl,N,EsSl<B,Hu,Nl,N,Es	B<Nl,N,EsGr,Hu,Sl<B,Nl,N,Es
Skipped breakfast ≥ 1/week (%)	40.8	27.2 ^̂	50.5	40.7 ^̂	42.6	41.6	19.7	14.8	23.5	17.1	56.1	42.9 ^̂̂	21.6	11.8 ^̂	Hu<,SlN,Es<B,Gr,Hu,SlNl<Gr,Hu,Sl	B<Gr,Hu,SlNl,N,Es<B,Gr,Hu,Sl

* P<0.05;

** p<0.01;

*** p<0.001 girls significantly lower than boys, adjusted for age;

‡ p<0.05.

‡‡ p<0.01;

‡‡‡ p<0.001 boys significantly lower than girls, adjusted for age;

^ p<0.05;

^̂ p<0.01;

^̂̂ P<0.001 high educated significantly lower than low educated, adjusted for age;

# p<0.05;

## p<0.01;

### p<0.001 lower educated significantly lower than higher educated, adjusted for age.

a pair wise comparison between countries using Bonferroni correction adjusted for age;

Mean number of days/week having breakfast ranged from 6.7 among Spanish girls to 5.1 among Slovenian girls. In Slovenia the highest prevalence (>50%) of skipping breakfast on one or more days per week was reported.

Children of lower educated parents reported less favorable intakes regarding soft drink, fruit juice, and breakfast than children of higher educated parents (Table 3 bottom half and Table S2), in the separate country analyses.

#### Physical activity behaviors

With an average of more than 40 minutes/week, children in Norway and Netherlands spent much more time cycling to school than children in other countries (Table 4 and Table S1). In Belgium, cycling is also a relevant active transportation mode. In general, girls cycled significantly fewer days per week to school than boys, but no significant gender differences in weekly minutes of cycling were found. Spanish and Norwegian girls reported the most minutes of walking to school. In general, girls reported more weekly minutes of walking to school than boys. No significant differences according to parental education in total active transport were observed (Table 5 and Table S2), however, differences were found in specific transport behaviors: children with low parental education were less likely to bike, but were more likely to walk to school.

**Table 4:** Means and standard deviations (SD) for physical activity in each of the participating countries, for boys and girls separately.

	Belgium		Greece		Hungary		Netherlands		Norway		Slovenia		Spain		Differences between countries a	
	(B)		(Gr)		(Hu)		(Nl)		(N)		(Sl)		(Es)			
Gender	Boys N = 477	Girls N = 516	Boys N = 500	Girls N = 586	Boys N = 459	Girls N = 563	Boys N = 456	Girls N = 463	Boys N = 450	Girls N = 518	Boys N = 570	Girls N = 601	Boys N = 493	Girls N = 529	Boys	Girls
Total active transport (days/week)	3.22.4	3.1±2.5	4.0±1.9	3.9±1.9	2.4±2.5	2.3±2.4	4.8±1.2	4.8±1.1	5.8±2.5‡‡	6.3±2.6	3.0±2.4	2.8±2.4	4.0±1.8	4.1±1.7	B,Sl<Gr,Nl,N,EsGr,Es<Nl,NHu<B,Gr,Nl,N,Sl,EsNl<N	B,Sl<Gr,Nl,N,EsGr,Es<Nl,NHu<B,Gr,Nl,N,Sl,EsNl,<N
Total active transport (min/week)	43±44	43±50	41±41	40±34	44±57	41±56	55±44	60±44	94±72‡‡‡	111±80	45±51	46±53	59±48	61±50	Gr,Hu<Nl,N,EsB,Sl<N,EsNl,Es<N	B,Gr,Hu,Sl<Nl,N,EsNl,Es<N
Active transport 24 h (min/day)	6.5±8.8	6.3±9.4	7.7±7.7	7.4±7.3	5.3±9.8	5.5±9.8	10.2±9.0	11.1±9.6	12.5±1	13.7±12	6.6±9.8	6.6±10	10.4±10	11.1±10	B,Gr,Sl,<Nl,N,EsHu<Gr,Nl,N,Sl,EsNl<N	B,Gr,Sl<Nl,N,EsHu<Gr,Nl,N,EsNl,Es<N
Cycling to school(days/week)	2.0±2.2	1.8±2.1	0.1±0.6	0 1±0.4	0.4±.2	0.2±0.8**	3.3±2.1	3.2±2.1	3.4±2.1	3.2±2.0	0.4±1.0	0.2±0.8 ***	0.1±0.4	0.0±0.1*	B<Nl,NGr,Es<B,Nl,N,SlHu,Sl<B,Nl,N	B<Nl,NGr,Hu,Sl,Es<B,Nl,N
Cycling to school(min/week)	27±39	25±41	1.5±12	0.3±2.6 *	6.3±24	3.8±18	42±47	46±50	46±45	49±48	6.7±23	4.6±21	0.8±7.4	0.2±1.9	B<Nl.NEs<B,NL,N,SlGr,Hu,Sl<B,Nl,N	B<Nl,NGr,Hu,Sl,Es<B,Nl,N
Walking to school (days/week)	1.3±2.0	1.3±2.0	3.9±1.9	4.0±1.9	2.0±2.3	2.1±2.3	1.5±2.1	1.6±2.1	2.5±2.2‡‡‡	3.1±2.1	2.5±2.3	2.6±2.3	3.9±1.8	4.1±1.8	Hu<Gr,N,Sl,EsB,Nl<Gr,Hu,N,Sl,EsN,Sl<Gr,Es	B,Nl<Gr,Hu,N,Sl,EsHu<Gr,N,Sl,EsN,Sl<Gr,Es
Walking to school (min/week)	16±30	18±35	40±36	39±34	38±53	37±53	13±22	14±22	48±56‡‡‡	62±60	38±46	42±50	58±47	61±50	B,Nl<Gr,Hu,N,Sl,EsGr,N,Sl<EsHu<N,Es	B,Nl<Gr,Hu,N,Sl,EsGr,Hu,Sl<N,Es
Sport participation (min/week)	229±148	191±145 ***	190±154	139±137 ***	282±179	236±161 ***	248±159	174±138 ***	310±166	238±154 ***	292±171	246±173 ***	249±166	148±139 ***	B<Hu,N,SlGr<B,Hu,Nl,N,Sl,EsNl,Es<N,SL	B,Nl<Hu,N,SlGr,Es<B,Hu,Nl,N,Sl

* P<0.05;

** p<0.01;

*** p<0.001 girls significantly lower than boys, adjusted for age;

‡ p<0.05;

‡‡ p<0.01;

‡‡‡ p<0.001 boys significantly lower than girls, adjusted for age;

a pair wise comparison between countries using Bonferroni correction adjusted for age.

**Table 5:** Means and standard deviations (SD) for physical activity behaviors in each of the participating countries, for children with low educated and high educated parents separately.

	Belgium		Greece		Hungary		Netherlands		Norway		Slovenia		Spain		Differences between countries a	
	(B)		(Gr)		(Hu)		(Nl)		(N)		(Sl)		(Es)			
Parental education	Low N = 105	High N = 561	Low N = 430	High N = 461	Low N = 320	High N = 443	Low N = 78	High N = 271	Low N = 183	High N = 533	Low N = 392	High N = 503	Low N = 171	High N = 708	Low	High
Total active transport (days/week)	3.4±2.3	3.0±2.4	4.1±1.8	3.8±2.0^Λ^	2.3±2.5	2.2±2.4	4.7±1.3	4.8±0.9	5.9±2.7	6.1±2.5	2.7±2.4	2.8±2.4	4.3±1.5 #	4.0±1.9	Sl<Gr,Nl,N,EsB<Nl,N,EsGr,Nl,Es<NHu<B,Gr,Nl,N,Es	B,Sl<Gr,Nl,N,EsGr,Nl<NHu<B,Gr,Nl,N,Sl,EsEs<Nl,N
Total active transport (min/week)	46±46	41±48	43±41	36±32^ΛΛ^	42±58	39±52	52±38	59±43	107±84	104±76	43±52	44±52	67±51	59±49 ^	B,Gr,Hu,Sl<N,EsNl,Es<N	B,Gr,Sl<Nl,N,EsHu<Nl,N,Sl,EsNl,Es<N
Active transport 24 h (min/day)	63.2±7.5	6.7±97	8.3±8.0	6.6±6.8^ΛΛ^	5.8±10.8	4.9±9.2	9.9±8.2	10.9±9.8	13.6±11.8	13.3±11.7	6.8±10.7	6.1±9.6	11.9±10	10.6±10	B,Gr<N,EsHu<Gr,Nl,N,EsSl<N,Es	B,Gr,Sl<Nl,N,EsHu<B,Gr,Nl,N,EsEs<N
Cycling to school(days/week)	23.0±2.2	1.8±2.1	0.1±0.5	0.1±0.5	0.3±1.1	0.2±0.9	2.8±2.2	3.2±2.1	3.2±2.1	3.4±2.0	0.3±0.8	0.3±0.9	0.1±0.4	0.0±0.3	B<Nl,NGr,Hu,Sl,Es<B,Nl,N	B<Nl,NEs<B,Nl,N,SlGr,Hu,Sl<B,Nl,N
Cycling to school(min/week)	26±37	27±42	0.8±1.0	0.5±3.9	5.9±24	4.1±18	37±45	44±49	51±47	49±48	4.0±18	6.1±23	0.8±8	0.5±5.0	B,Nl<NGr,Hu,Sl,Es<B,Nl,N	B<Nl,NEs<B,Nl,N,SlGr,Hu,Sl<B,Nl,N
Walking to school (days/week)	1.4±2.1	1.2±1.9	4.0±1.8	3.7±2.0^Λ^	2.0±2.3	2.0±2.3	2.0±2.3	1.6±2.1	2.7±2.2	2.7±2.2	2.4±2.3	2.4±2.3	4.3±1.5	3.9±1.9 ^	B,<Gr,N,Sl,EsHu<Gr,N,EsNl,N,Sl<Gr,Es	B<Gr,Hu,N,Sl,EsHu,Nl<Gr,N,Sl,EsN,Sl<Gr,Es
Walking to school (min/wk)	19±35	14±30	43±38	36±32^ΛΛ^	36±53	34±50	15±17	14±21	56±62	55±59	39±50	38±47	66±50	58±48 ^	B,Nl<Gr,Hu,N,Sl,EsGr,Hu,Sl<N,Es	B,Nl<Gr,Hu,N,Sl,EsGr,Hu,Sl<N,Es
Sport participation (min/week)	187±148	212±144	144±146 ###	183±145	234±169 ###	282±171	225±165	205±136	240±160 ##	283±160	258±173	274±170	173±163#	207±158	Es<Hu,N,SlB<SlGr<Hu,Nl,N,Sl	B,Gr,Nl,Es<Hu,N,Sl

ΛΛΛ P<0.001 high educated significantly lower than low educated, adjusted for age;

# p<0.05;

## p<0.01;

### p<0.001 lower educated significantly lower than higher educated, adjusted for age.

a pair wise comparison between countries using Bonferroni correction adjusted for age.

For engagement in sport activities, boys reported on average 260 minutes/week ranging from more than 300 minutes/week in Norway to less than 200 minutes/week in Greece. Girls reported on average almost 200 minutes/week, ranging from 250 minutes/week in Slovenia to less than 150 minutes/week in Greece. Girls reported lower engagement in sport across all countries. Children of higher educated parents participated significantly more in sports than those from lower educated parents.

#### Sedentary behaviors and sleeping

Across the countries boys reported spending about 2½ hours and girls somewhat less than 2 hours on screen-viewing activities (TV and computer-time combined) in 24-h recall; girls in Spain reported the lowest screen time (table 6). In all countries mean total screen time, as well as TV and computer time were higher for boys than girls. The results based on the frequency questions were similar although somewhat higher. Again lower 24-h recall results may be due to the fact that questionnaire administration was not done on Mondays, therefore excluding weekend days (often with more time spend in screen activities) from the 24-h recall assessment. Children of higher educated parents reported less screen time than those from lower educated parents: less time watching TV, using the computer, as well as total screen time (table 6 bottom half and Table S2).

**Table 6:** Means and standard deviations (SD) for sedentary behaviors in each of the participating countries, for boys and girls separately and for children with low educated and high educated parents separately.

	Belgium		Greece		Hungary		Netherlands		Norway		Slovenia		Spain		Differences between countries a	
	(B)		(Gr)		(Hu)		(Nl)		(N)		(Sl)		(Es)			
Gender	Boys N = 477	Girls N = 516	Boys N = 500	Girls N = 586	Boys N = 459	Girls N = 563	Boys N = 456	Girls N = 463	Boys N = 450	Girls N = 518	Boys N = 570	Girls N = 601	Boys N = 493	Girls N = 529	Boys	Girls
Screen time FQ (min/day)	205±102	178±95 ***	214±103	179±85 ***	233±108	198±103 ***	223±115	185±101 ***	196±97	168±91 ***	213±112	174±103 ***	193±102	160±90 ***	N,Es<Gr,Hu,Nl,Sl	N<B,Gr,Hu,Nl,SlEs<,Gr,Hu
Screen time -24 h recall (min/day)	124±92	107±83 **	155±103	122±87 ***	166±113	131±100 ***	153±105	112±88 ***	132±95	101±80 ***	131±104	100±88 ***	122±95	89±73 ***	B,N,Es<Gr,Hu,NlSl<Gr,Hu	B<GrEs<B,Gr,Hu,NlSl,N<Gr,Hu
TV time FQ(min/day)	116±61	110±63	126±61	120±56	123±62	116±61	116±65	104±60 **	105±56	97±54 *	120±65	108±64 **	109±56	97±54 **	Nl<GrN<B,Gr,Hu,Nl,SlEs<Gr,Sl	B,Hu,Nl,Sl<GrN<B,Gr,Hu,SlEs<B,Gr,Sl
TV time 24 h recall (min/day)	78±57	77±60	99±63	89±62 **	90±63	85±62	83±64	67±54 ***	72±58	62±50 **	78±63	68±60 **	77±54	64±52 ***	B,Hu,Nl,Sl,Es<GrN<Gr,Hu	B,Hu,Sl<GrNl,Es<B,Gr,HuN<B,Gr,Hu,Sl
Computer time Q (min/day)	89±62	69±53***	88±61	60±51 ***	110±66	82±60 ***	106±66	81±59 ***	91±60	71±53 ***	93±65	64±57 ***	85±61	6350 ***	N,Es<Hu,NlB<Nl	Gr,Es<Hu,NlN,Sl<Nl
Computer time- 24 h recall (min/day)	47±60	29±56 ***	55±63	33±48 ***	75±73	46±59 ***	71±65	45±56 ***	60±61	40±48 ***	52±64	33±47 ***	45±57	25±39 ***	Es<HuB<Hu,NlN,Sl<Nl	B<Hu,NlSl<NlEs<Hu.Nl.N
Sleeping habits(hr/night)	9.6±0.7	9.7±0.8	8.7±0.8‡‡	8.8±0.8	9.0±0.7	9.1±0.7	9.5±0.8	9.7±0.8	9.2±0.6	9.2±0.6	9.1±0.7	9.1±0.7	9.3±0.7‡	9.4±0.7	Gr<B,Hu,Nl,N,Sl,EsN,Es<B,NlSl,Hu<B,Nl,N,Es	Gr<B,Hu,Nl,N,Sl,EsHu<B,NLEsN,Es<Bl,NSl<B,Nl,N,Es
**Parental education**	**Low N = 105**	**High N = 561**	**Low N = 430**	**High N = 461**	**Low N = 320**	**High N = 443**	**Low N = 78**	**High N = 271**	**Low N = 183**	**High N = 533**	**Low N = 392**	**High N = 503**	**Low N = 171**	**High N = 708**	**Low**	**High**
Screen time FQ (min/day)	184±90	181±93	201±98	190±87	230±110	198±100 ^̂̂	224±123	177±106̂̂	184±95	174±90	208±111	175±101 ^̂̂	191±106	171±92̂	N<Hu,Nl,Sl	N<B,Gr,HuEs<Gr
Screen time −24 h recall (min/day)	115±96	109±81	142±98	130±93	162±109	125±98 ^̂̂	162±125	112±92^̂	116±91	109±80	126±98	95±82 ^̂̂	121±92	100±82 ^̂	N<Gr,Hu,Nl	B,Hu,Nl,N,Sl,Es<Gr
TV time FQ(min/day)	105±63	111±61	130±58	119±55^̂	130±65	112±60^̂̂	124±65	96±61^̂	103±53	97±52	121±65	106±60^̂	108±59	102±53	B,Es<GrN<Gr,Hu,,Sl	Nl,<B,GrN<B,Gr,Hu,Sl,Hu,Sl,Es<Gr
TV time 24 h recall (min/day)	72±59	76±56	98±61	88±61̂	99±64	75±58̂̂̂	90±65	65±57̂̂	68±49	63±51	79±61	63±56 ^̂̂	76±54	69±52	B,N<Gr,HuSl,Es<Gr	B,Hu,Nl,Es<GrSl<B,GrN<B,Gr,Es
Computer time FQ (min/day)	80±60	71±52	71±59	72±52	101±66	87±59^̂	109±72	80±59^̂	80±58	77±54	86±64	68±58 ^̂̂	83±62	70±53 ^̂	Gr<Hu,Nl,SlN<Hu,Nl	
Computer −24 h recall (min/day)	44±61	34±49	44±560	41±53	62±70	51±59^	72±75	47±54^̂	48±57	46±51	46±57	32±48 ^̂̂	44±59	32±46 ^̂	Gr,N<Nl	Es<Gr,Hu,Nl,NB,Sl<Nl
Sleeping habits(hr/night)	9.5±0.8#	9.7±0.7	8.8±0.8	8.7±0.7^	9.1±0.8	9.1±0.7	9.5±0.9	9.7±0.7	9.2±0.7	9.2±0.6	9.2±0.8	9.1±0.7	9.4±0.8	9.3±0.6	Gr<B,Hu,Nl,N,Sl,EsHu,Sl<B,Nl	Gr<B,Hu,Nl,N,Sl,EsHu,N,Es<B,NlSl<B,Nl,N,Es

* P<0.05;

** p<0.01;

*** p<0.001 girls significantly lower than boys, adjusted for age;

‡ p<0.05;

‡‡ p<0.01;

‡‡‡ p<0.001 boys significantly lower than girls, adjusted for age;

^ p<0.05;

^̂ p<0.01;

^̂̂ P<0.001 high educated significantly lower than low educated, adjusted for age;

# p<0.05;

## p<0.01;

### p<0.001 lower educated significantly lower than higher educated, adjusted for age.

a pair wise comparison between countries using Bonferroni correction adjusted for age.

Parents of boys and girls reported on average that their child slept 9.2 hours/night. This varied between 8.7 hours for Greek boys to 9.7 hours for Belgian girls. Significantly less hours of sleep were reported for Greek boys and girls than in all the other countries (Table 6 and Table S1). No significant differences in sleeping behavior were reported between boys and girls or between high and lower educated parents (Table 6 and Table S2).

## Discussion

The prevalence of measured overweight -including obesity- across seven countries from different regions in Europe was 25.8% and 21.8% (IOTF) or 34.6% and 26.8% (WHO) for boys and girls respectively. The patterns in differences in overweight and obesity were similar for IOTF and WHO criteria, but WHO criteria resulted in somewhat to substantially higher prevalence rates. The prevalence of overweight was much higher than reported for the same seven countries in the Health Behavior in School-aged Children (HBSC) report [33]. HBSC relied, however, on self-reported weight and height, and the data are between 4–5 years older. Our finding that overweight prevalence as well as engagement in some of the ‘risk’ behaviors was higher in the included countries in southern and central European region than in the countries located in the Northwest of Europe is in line with other studies including HBSC [7]. It may be that the overweight rise is ‘leveling off’ in some countries [34], but the present prevalence remains higher than desirable and unacceptably high in some countries. Although the overweight prevalence in Greek boys of more than 40% is especially worrisome, the prevalence in all countries calls for action, especially when the results regarding the EBRB are taken into account.

Across all countries children engage frequently in dietary, (lack of) physical activity and sedentary behaviors that are regarded as potential risk behaviors for becoming overweight obese, with large differences between countries. Many children skipped breakfast on one or more days per week, especially in Greece and Slovenia, and the mean intakes of sugar-sweetened beverages in the Netherlands, Hungary and Slovenia was high. Low levels of active transport were reported especially in Belgium, Slovenia and Hungary, while low levels of sports were reported in Greece. Norwegian children reported the most minutes of cycling and walking to school. This is probably partly due to over-reporting due to the large seasonal differences in Norway. Norwegian children tend to cycle to school in summer, and walk to school in winter [35], and they probably reported their usual pattern for both seasons. Also in other countries some children reported active transport activities to school on more than five times per week. It may be that these children cycled to school and walked back, commuted to school more than once per day, or had other valid reasons, so total active transport time needs to be interpreted with caution.

For sedentary behaviors, screen activities were high in all countries, wherein children spent on average more than 2 hours/day in TV and computer activities. The results also indicate that children’s screen time is quite close to being equally divided between TV and other screen-viewing behaviors, confirming that sedentary behavior interventions should not be restricted to TV time only.

Furthermore, we found that overweight and participation in the potential risk behavior was more prevalent in boys than girls, with the exception of sports activities. In addition, overweight and potential risk behaviors are generally more likely in children of parents with fewer years of formal education. The difference in prevalence of overweight and risk behaviors according to parental education in Slovenia appeared to be especially worrisome and the educational disparities and differences in these disparities across Europe requires further research. In-depth analyses (not presented in this study) showed that these patterns were similar for both boys and girls. This supports earlier studies [7] indicating that interventions aimed at preventing overweight and obesity in school-aged children should pay specific attention to boys and children of low educated parents.

The study has some limitations. First, differences in response rates at school and student level were apparent, which may have reduced the external validity of the findings. The fact that schools were especially difficult to recruit in the Netherlands is in line with other school-based research in this country in recent years. There is a great emphasis on youth health research. Some school-based research is obligatory (i.e. as conducted by municipal health services [36] and participation in many additional school-based research is requested. Schools are therefore more and more reluctant to participate. Although the Dutch results are in line with other data regarding overweight and obesogenic behaviors in this age group in the Netherlands [37], [38] the Dutch data should be interpreted with extra caution. The fact that response rates at the child level were lower in Hungary, Norway and Spain was mostly because parents did not provide active parental informed consent. This may have resulted in participation of children from parents who are more interested in issues regarding obesity prevention, and thus to biased results. Parental data in the Netherlands might be biased to higher levels of education, which may have resulted in lower overall levels of overweight and obesity for this country.

Furthermore, data on dietary, physical activity and sedentary behavior were based on self-reports, and thus possibly biased. Nevertheless, the measures showed good test-retest reliability and construct validity [30], and for some behaviors both 24-h recall and frequency questions were included- that showed similar results. The intraclass coefficients for the 24-h recall questions were lower than for the frequency questions; this was to be expected because behavior in the last 24 hours (24-h recall) is likely to vary more than usual behavior as assessed by the frequency questions. Further, in the present study the measure of physical activity behavior did not capture participation in all physical activities, and only included measures of active transport and sports participation. Additionally, active transport was restricted to transport to and from schools. This means that our results do not represent total active travel. It may, however, be likely that children who use active transportation to school may also be more likely to go by bike or foot to other destinations. Future research will be conducted to examine which behavioral, cognitive, and environmental factors are associated with the differences in overweight indices and EBRBs across and within the countries. Finally, the potential risk behaviors included in the present study are not proven causes of adiposity among schoolchildren. The evidence regarding these potential risk behaviors is largely based on observational research that only allow conclusions regarding associations rather than causation.

Strengths of the present study include the large multinational sample from different regions across Europe, the standardized data collection protocol across the different countries, measured weight, height and waist circumference, and the inclusion of a large range of potential EBRBs, including sedentary behaviors and sleep duration.

In conclusion, prevalence of overweight is high across Europe among school-aged children, with especially worrisome prevalence in Greece. Many children engage frequently in dietary, physical activity and sedentary behaviors that are associated with the likelihood of becoming overweight and obese, with large differences in such behaviors between countries and region. Region or country-specific policies and interventions are needed to contribute to curbing the overweight epidemic in European schoolchildren.

## Supporting Information

Table S1 **Medians and 25–75% percentiles for dietary, physical activity and sedentary behaviors in each of the participating countries, for boys and girls.**
(DOC)

Table S2 **Medians and 25–75% quartiles for dietary, physical activity and sedentary behaviors in each of the participating countries, for children with low educated and high educated parents.**
(DOC)

## References

[1] Lean M, Crawford D, Jeffery R, Ball K, Brug J (2010). Health consequences of overweight and obesity in adults.. Obesity epidemiology. Oxford, UK: Oxford University Press.

[2] Reilly J, Houston- Callaghan K, Donaghey Z, Hammed S, Crawford D, Jeffery RW, Ball K, Brug J (2010). Physical health consequences of child and adolescent obesity.. Obesity epidemiology.Oxford, UK: Oxford University Press.

[3] Serdula MK, Ivery D, Coates RJ, Freedman DS, Williamson DF (1993). Do obese children become obese adults? A review of the literature.. Preventive Medicine.

[4] Singh AS, Mulder C, Twisk JW, van Mechelen W, Chinapaw MJM (2008). Tracking of childhood overweight into adulthood: a systematic review of the literature.. Obesity Reviews.

[5] International Association for the Study of Obesity (2012). http://www.iaso.org/publications/trackingobesity/under-data-down-loads/Accessed.

[6] Lien N, Henriksen HB, Nymoen LL, Wind M, Klepp KI (2010). Availability of data assessing the prevalence and trends of overweight and obesity among European adolescents.. Public Health Nutrition.

[7] Haug E, Rasmussen M, Samdal O, Iannotti R, Kelly C (2009). Overweight in school-aged children and its relationship with demographic and lifestyle factors: results from the WHO-Collaborative Health Behaviour in School-aged Children (HBSC) study.. International Journal of Public Health.

[8] Yngve A, De Bourdeaudhuij I, Wolf A, Grjibovski A, Brug J (2008). Differences in revalence of overweight and stunting in 11-year olds across Europe: The Pro Children Study.. European Journal of Public Health.

[9] Cappuccio FP, Taggart FM, Kandala NB, Currie A, Peile E (2008). Meta-analysis of short sleep duration and obesity in children and adults.. Sleep.

[10] Chen X, Beydoun MA, Wang Y (2008). Is sleep duration associated with childhood obesity? A systematic review and meta-analysis.. Obesity (Silver Spring).

[11] Maffeis C (2000). Aetiology of overweight and obesity in children and adolescents.. European Journal of Pediatrics.

[12] Rennie KL, Johnson L, Jebb SA (2005). Behavioural determinants of obesity.. Best Practice Research in Clinical Endocrinol Metabolism.

[13] Swinburn BA, Caterson I, Seidell JC, James WP (2004). Diet, nutrition and the prevention of excess weight gain and obesity.. Public Health Nutrition.

[14] Douthwaite W, Summerbell C, Moore H (2012). http://www.projectenergy.eu/oeffentlicher_bereich/publications/reports/WP2-Phase%201%20Report%20def.pdf.

[15] Fredriks AM, Van Buuren S, Sing RA, Wit JM, Verloove-Vanhorick SP (2005). Alarming prevalences of overweight and obesity for children of Turkish, Moroccan and Dutch origin in The Netherlands according to international standards.. Acta Paediatrica.

[16] Gordon-Larsen P, Adair LS, Popkin BM (2003). The relationship of ethnicity, socioeconomic factors, and overweight in US adolescents.. Obesity Research.

[17] Lobstein T, Frelut ML (2003). Prevalence of overweight among children in Europe.. Obesity Reviews.

[18] Brug J, Lien N, Klepp KI, van Lenthe FJ (2010). Exploring overweight, obesity and their behavioural correlates among children and adolescents: results from the Health-promotion through Obesity Prevention across Europe project.. Public Health Nutrition.

[19] Delva J, O’Malley PM, Johnston LD (2006). Racial/ethnic and socioeconomic status differences in overweight and health-related behaviors among American students: national trends 1986–2003.. Journal of Adolescent Health.

[20] Gorely T, Marshall SJ, Biddle SJ (2004). Couch kids: correlates of television viewing among youth.. International Journal of Behavioral Medicine.

[21] Sallis JF, Prochaska JJ, Taylor WC (2000). A review of correlates of physical activity of hildren and adolescents.. Medicine and Science in Sports and Exercise.

[22] te Velde SJ, Wind M, van Lenthe FJ, Klepp KI, Brug J (2006). Differences in fruit and vegetable intake and determinants of intakes between children of Dutch origin and non-Western ethnic minority children in the Netherlands - a cross sectional study.. International Journal of Behavioral Nutrition and Physical Activity.

[23] te Velde SJ, De Bourdeaudhuij I, Thorsdottir I, Rasmussen M, Hagstromer M, et al. 2007) Patterns in sedentary and exercise behaviors and associations with overweight in 9–14-year-old boys and girls–a cross-sectional study.. BMC Public Health.

[24] Van Der Horst K, Chinapaw MJ, Twisk JW, Van Mechelen W (2007). A brief review on correlates of physical activity and sedentariness in youth.. Medicine and Science in Sports and Exercise.

[25] Wang Y, Liang H, Tussing L, Braunschweig C, Caballero B (2007). Obesity and related isk factors among low socio-economic status minority students in Chicago.. Public Health utrition.

[26] Bleich S, Ku R, Wang Y (2011). Relative contribution of energy intake and energy expenditure to childhood obesity: a review of the literature and directions for future research.. International Journal of Obesity.

[27] Brug J, te Velde SJ, Chinapaw MJ, Bere E, De Bourdeaudhuij I (2010). Evidence-based development of school-based and family-involved prevention of overweight across Europe: the ENERGY-project’s design and conceptual framework.. BMC Public Health.

[28] van Stralen M, te Velde S, Singh A, De Bourdeaudhuij I, Martens M (2011). EuropeaN Energy balance Research to prevent excessive weight Gain among Youth (ENERGY) project: Design and methodology of the ENERGY cross-sectional survey.. BMC Public Health.

[29] Klepp KI, Perez-Rodrigo C, De Bourdeaudhuij I, Due PP, Elmadfa I (2005). Promoting fruit and vegetable consumption among European schoolchildren: rationale, conceptualization and design of the pro children project.. Annals of Nutrition and Metabolism.

[30] Singh AS, Vik FN, Chinapaw MJ, Uijtdewilligen L, Verloigne M (2011). Test-retest reliability and construct validity of the ENERGY-child questionnaire on energy balance-related behaviours and their potential determinants: the ENERGY-project.. Int J Behav Nutr Phys Act.

[31] Cole TJ, Bellizzi MC, Flegal KM, Dietz WH (2000). Establishing a standard definition for child overweight and obesity worldwide: international survey.. BMJ.

[32] de Onis M, Onyango AW, Borghi E, Siyam A, Nishida C (2007). Development of a WHO growth reference for school-aged children and adolescents.. Bulletin of the World Health Organization.

[33] Currie C, Gabhainn SN, Godeau E, Roberts C, Smith R (2008). Inequalities in young people’s health..

[34] Rokholm B, Baker J, Sørensen T (2010). The leveling off of the obesity epidemic since the year 1999 - a review of evidence and perspectives.. Obesity Reviews.

[35] Børrestad L, Andersen L, Bere E (2011). Seasonal and socio-demographic determinants of school commuting.. Preventive Medicine.

[36] Jansen W, Raat H, Zwanenburg EJ, Reuvers I, van Walsem R (2008). A school-based intervention to reduce overweight and inactivity in children aged 6–12 years: study design of a randomized controlled trial.. BMC Public Health.

[37] Jansen W, Mackenbach JP, Joosten-van Zwanenburg E, Brug J (2010). Weight status, energy-balance behaviours and intentions in 9–12-year-old inner-city children.. Journal of Human Nutrition and Dietetics.

[38] Centraal Bureau voor de Statistiek (CBS) [Statistics Netherlands] (2010). Trendrapport 2010: Landelijke Jeugdmonitor *[Trend report 2010: National Youth Monitor]*..

