# Supplementary material for: Differences in Weight Status and Energy-Balance Related Behaviors among Schoolchildren across Europe: The ENERGY-Project
Source: PLoS One. 2012 Apr 25;7(4):e34742. doi: 10.1371/journal.pone.0034742 (PMC3338827; doi:10.1371/journal.pone.0034742)
Supplement: Table S1 — Medians and 25–75% percentiles for dietary, physical activity and sedentary behaviors in each of the participating countries, for boys and girls. (DOC) [file pone.0034742.s001.doc]

Table S1: Medians and 25-75% percentiles for dietary, physical activity and sedentary behaviors in each of the participating countries, for boys and girls separately

|  | **Belgium**  **(B)** | | **Greece**  **(Gr)** | | **Hungary**  **(Hu)** | | **Netherlands**  **(Nl)** | | **Norway**  **(N)** | | **Slovenia**  **(Sl)** | | **Spain**  **(Es)** | |
| --- | --- | --- | --- | --- | --- | --- | --- | --- | --- | --- | --- | --- | --- | --- |
|  | **Boys**  **N=477** | **Girls**  **N=516** | **Boys**  **N=500** | **Girls**  **N=586** | **Boys**  **N=459** | **Girls**  **N=563** | **Boys**  **N=456** | **Girls**  **N=463** | **Boys**  **N=450** | **Girls**  **N=518** | **Boys**  **N=570** | **Girls**  **N=601** | **Boys**  **N=493** | **Girls**  **N=529** |
| **Dietary behaviors** |  |  |  |  |  |  |  |  |  |  |  |  |  |  |
| Soft drink FFQ (ml/day) | 249 (74; 750) | 214 (41;570) | 47 (24; 141) | 36 (18; 107) | 429 (102;883) | 261 (71;750) | 500 (196;1000) | 500 (107;797) | 154 (71;356) | 95 (36;215) | 166 (41;570) | 71 (18;250) | 107 (41;259) | 47 (18;119) |
| Soft drink-24h recall (ml/day) | 250 (0;500) | 250 (0; 500) | 0 (0;330 | 0 (0; 250) | 500 (0;1000) | 500 (0;1000) | 500 (250;1000) | 500 (250;750) | 0 (0;500) | 0(0;250) | 250 (0;750) | 250 (0;500) | 0 (0;330) | 0 (0;250) |
| Fruit Juice FFQ (ml/day) | 196 (36;393) | 196 (36; 321) | 214 (107;381) | 214 (107;250) | 214 (41;463) | 214 (59;463) | 249 (83;520) | 249 (107;565) | 107 (36;321) | 107 (18;214) | 250 (83;580) | 249 (71;500) | 196 (71;330) | 107 (47;250) |
| Fruit juice-24h recall (ml/day) | 250 (0;500) | 250 (0; 500) | 250 (0; 330) | 250 (0;500) | 250 (0;500) | 250 (0; 500) | 250 (0;580) | 250 (0;500) | 0 (0;250) | 0 (0;250) | 250 (0;580) | 250 (0;500) | 250 (0;250) | 250 (0;250) |
| Breakfast (days/week) | 7 (6;7) | 7 (6; 7) | 7 (4;7) | 7 (4;7) | 7 (5;7) | 7 (5;7) | 7 (7;7) | 7 (7;7) | 7 (7;7) | 7 (7;7) | 6 (3;7 | 6 (3;7) | 7 (7;7) | 7 (7;7) |
| Skipped breakfast ≥ 1/week (%) | 30.7 | 30.5 | 48.0 | 46.1 | 38.4 | 47.5 | 24.0 | 22.0 | 22.0 | 20.2 | 51.9 | 51.4 | 17.0 | 12.0 |
| **Physical activity behaviors** | |  |  |  |  |  |  |  |  |  |  |  |  |  |
| Total active transport (days/week) | 5 (1;5) | 4 (0;5) | 5 (4;5) | 5 (3;5) | 2 (0;5) | 1 (0;5) | 5 (5;5) | 5 (5;5) | 5 (5;7) | 5 (5;9) | 4 (0;5) | 3 (0;5) | 5 (4;5) | 5 (4;5) |
| Total active transport (min/ week) | 30 (6;78) | 30 (0; 78) | 30 (30;54) | 30 (30;49) | 30 (0;80) | 26 (0;80) | 30 (30;80) | 36 (30;80) | 80 (36;130) | 92 (48;160) | 30 (0;80) | 30 (0;80) | 32 (30;80) | 30 (30;80) |
| Active transport 24h recall (min/day) | 6 (0;6) | 0 (0;6) | 6 (6;6) | 6 (0;6) | 0 (0;6) | 0 (0;6) | 6 (6;16) | 6 (6;16) | 6 (6;16) | 16 (6;26) | 0 (0;6) | 0 (0;6) | 6 (0;16) | 6 (6;16) |
| Cycling to school  (days /week) | 1 (0;5) | 1 (0;4) | 0 (0;0) | 0 (0;0) | 0 (0;0) | 0 (0;0) | 5 (1;5) | 5 (1;5) | 5 (1;5) | 4 (1;5) | 0 (0;0) | 0 (0;0) | 0 (0;0) | 0 (0;0) |
| Cycling to school  (min/week) | 12 (0;30) | 6 (0;30) | 0 (0;0) | 0 (0;0) | 0 (0;0) | 0 (0;0) | 30 (6;80) | 30 (6;80) | 30 (10.5;80) | 30(6;80) | 0 (0;0) | 0 (0;0) | 0 (0;0) | 0 (0;0) |
| Walking to school (days/week) | 2 (0;2) | 0 (0;2) | 5 (4;5) | 5 (4;5) | 1 (0;5) | 1 (0;5) | 0 (0;40) | 0 (0;4) | 2 (0;5) | 4 (1;5) | 3 (0;5) | 3 (0;5) | 5 (3;5) | 5 (4;5) |
| Walking to school (min/week) | 2 (0;30) | 0 (0;29) | 30 (30;44) | 30 (30;44) | 6 (0;64) | 16 (0;80) | 0 (0;26) | 0 (0;30) | 30 (0;80) | 40 (12;80) | 30 (0;79) | 30 (0;80) | 32 (30;80) | 30 (30;80) |
| Sport participation (min/week) | 210 (120;330) | 150 (90;270) | 180 (0;300) | 180 (0;300) | 300 (120;420) | 210 (120;330) | 240 (120;330) | 150 (60;240) | 300 (180;420) | 240 (120;330) | 300 (150;420) | 240 (120;360) | 240 (120;360) | 120 (0;240) |
| **Sedentary behavior** |  |  |  |  |  |  |  |  |  |  |  |  |  |  |
| Screen time FQ (min/day) | 195 (133;270) | 159 (103;244) | 201 (137;283) | 171 (120;233) | 227 (146;296) | 184 (120;266) | 210 (137;309) | 159 (107;249) | 178 (124;257) | 150 (106;210) | 193 (129;279) | 161 (95;236) | 171 (118;249) | 137 (95;206) |
| Screen time -24h recall (min/day) | 120 (60;180) | 90 (60;150) | 150 (60;210) | 90 (60;180) | 150 (90;240) | 120 (60;180) | 120 (60;210) | 90 (60;150) | 120 (60;180) | 90 (60;120) | 120 (60;180) | 90 (30;150) | 90 (60;180) | 60 (30;120) |
| TV time FQ(min/day) | 111 (69;159) | 99 (60;154) | 120 (77;167) | 120 (77;159) | 116 (77;168) | 107 (69;159) | 109 (64; 159) | 90 (60;137) | 99 (69;137) | 81 (60;129) | 107 (69;163) | 99 (60;150) | 99 (69;141) | 86 (60;129) |
| TV time 24h recall (min/day) | 60 (30;120) | 60 (30; 120) | 90 (60;143) | 60 (60;120) | 90 (60;120) | 60 (30;120) | 60 (30;120) | 60 (30;90) | 60 (30;90) | 60 (30;90) | 60 (30;120) | 60 (30;90) | 60 (30;120) | 60 (30;90) |
| Computer time FQ (min/day) | 73 (39;128) | 60 (30;99) | 77 (39;120) | 51 (21;86) | 99 (60;154) | 69 (34;116) | 94 (54; 150) | 60 (30;111) | 77 (43;129) | 60 (30;99) | 77 (39; 137) | 51 (30;90) | 69 (39;119) | 47 (30;81) |
| Computer time- 24h recall (min/day) | 30 (0;60) | 0 (0;30) | 30 (0;90) | 0 (0;60) | 60 (0;120) | 30 (0;60) | 60 (30;120) | 30 (0;60) | 30 (0;90) | 30 (0;60) | 30 (0;90) | 30 (0;60) | 30 (0;60) | 0 (0;30) |
| Sleeping habits(hr/night) | 9.7 (9.0;10.0) | 9.7 (9;10) | 8.6 (8.3;9.3) | 8.6 (8.3;9.3) | 9.0 (8.6; 9.3) | 9.0 (8.6;9.3) | 9.7 (9.0;10.0) | 9.7(9.0;10.0) | 9.3 (8.7;9.7) | 9.3 (8.7;9.6) | 9.0 (8.7;9.6) | 9 (8.6; 9.6) | 9.3 (9.0;9.9) | 9.3 (9.0;10.0) |
