# Supplementary material for: Differences in Weight Status and Energy-Balance Related Behaviors among Schoolchildren across Europe: The ENERGY-Project
Source: PLoS One. 2012 Apr 25;7(4):e34742. doi: 10.1371/journal.pone.0034742 (PMC3338827; doi:10.1371/journal.pone.0034742)
Supplement: Table S2 — Medians and 25–75% quartiles for dietary, physical activity and sedentary behaviors in each of the participating countries, for children with low educated and high educated parents. (DOC) [file pone.0034742.s002.doc]

Table S2: Medians and 25-75% quartiles for dietary, physical activity and sedentary behaviors in each of the participating countries, for children with low educated and high educated parents separately.

|  | **Belgium**  **(B)** | | **Greece**  **(Gr)** | | **Hungary**  **(Hu)** | | **Netherlands**  **(Nl)** | | **Norway**  **(N)** | | **Slovenia**  **(Sl)** | | **Spain**  **(Es)** | |
| --- | --- | --- | --- | --- | --- | --- | --- | --- | --- | --- | --- | --- | --- | --- |
|  | **Low (N=105)** | **High (n=561)** | **Low (n=430)** | **High (n=461)** | **Low (n=320)** | **High (n=443)** | **Low (n=78)** | **High (n=271)** | **Low (n=182)** | **High (n=533)** | **Low (n=391)** | **High (N=503)** | **Low (n=171)** | **High (N=708)** |
| **Dietary behaviors** |  |  |  |  |  |  |  |  |  |  |  |  |  |  |
| Soft drink FFQ (ml/day) | 393 (107;990) | 141 (41;500) | 47 (24;119) | 36 (18;83) | 429 (107;1000) | 248 (71;694) | 391 (214; 835) | 321 (107;750) | 119 (59;321) | 107 (41; 249) | 107 (36;429) | 83 (18;321) | 83 (36;273) | 54 (24;154) |
| Soft drink-24h recall (ml/day) | 330 (0;750) | 250 (0;500) | 0 (0;250) | 0 (0;250) | 500 (250;1000) | 500 (0;1000) | 500 (250; 1000) | 500 (250;750) | 250 (0;500) | 0 (0; 250) | 250 (0; 750) | 250 (0; 500) | 0 (0; 330) | 0 (0; 250) |
| Fruit Juice FFQ (ml/day) | 214 (41;463) | 196 (36;252) | 196 (94;330) | 214 (107; 259) | 249 (59;463) | 214 (54;463) | 290 (80; 724) | 214 (41;497) | 107 (18;214) | 107 (36; 250) | 250 (71; 580) | 214 (71; 500) | 214 (107; 356) | 141 (47; 250) |
| Fruit juice-24h recall (ml/day) | 250 (0;500) | 250 (0;250) | 250 (0;250) | 250 (0;250) | 250 (0;580) | 250 (0;500) | 415 (0; 770) | 250 (0; 500) | 0 (0;250) | 0 (0;250) | 250 (0;580) | 250 (0; 500) | 250 (0; 250) | 250 (0; 250) |
| Breakfast (days/week) | 7 (5;7) | 7 (6; 7) | 6 (4;7) | 7 (5; 7) | 7 (5; 7) | 7 (5; 7) | 7 (7; 7) | 7 (7; 7) | 7 (7; 7) | 7 (7; 7) | 6 (2; 7) | 7 (4; 7) | 7 (6;7) | 7 (7; 7) |
| Skipped breakfast ≥ 1/week (%) | 40.8 | 27.2 | 50.5 | 40.7 | 42.6 | 41.6 | 19.7 | 14.8 | 23.5 | 17.1 | 56.1 | 42.9 | 21.6 | 11.8 |
| **Physical activity behaviors** | |  |  |  |  |  |  |  |  |  |  |  |  |  |
| Total active transport (days/week) | 5 (1; 5) | 3 (0;5) | 5 (4.5; 5) | 5 (2;5) | 1 (0; 5) | 1 (0;5) | 5 (5; 5) | 5 (5;5) | 5 (5; 8) | 5 (5;8) | 3 (0; 5) | 3 (0;5) | 5 (5; 5) | 5 (4;5) |
| Total active transport (min/ week) | 30 (17; 80) | 30 (0; 61) | 30 (30; 80) | 30 (24;30) | 16 (0; 80) | 16 (0;78) | 30 (30;80) | 36 (30;80) | 80 (36; 160) | 82 (46;130) | 30 (0; 80) | 30 (0; 80) | 71 (30; 80) | 30 (30;80) |
| Active transport 24h recall (min/day) | 6 (0; 11) | 0 (0;6) | 6 (6; 18) | 6 (0;6) | 0 (0; 6) | 0 (0;6) | 6 (6; 16) | 6 (6;16) | 16 (6; 16) | 12 (6;19) | 0 (0; 6) | 0 (0;6) | 6 (6; 16) | 6 (0;16) |
| Cycling to school  (days /week) | 1 (0; 5) | 1 (0;4) | 0 (0; 0) | 0 (0;0) | 0 (0; 0) | 0 (0;0) | 3.5 (0; 5) | 4 (1;5) | 4 (1; 5) | 5 (2;5) | 0 (0; 0) | 0 (0;0) | 0 (0; 0) | 0 (0; 0) |
| Cycling to school  (min/week) | 6 (0; 30) | 6 (0;30) | 0 (0; 0) | 0 (0;0) | 0 (0; 0) | 0 (0;0) | 30 (0; 67.5) | 30 (6;80) | 30 (6; 80) | 30 (12;80) | 0 (0; 0) | 0 (0;0) | 0 (0; 0) | 0 (0;0) |
| Walking to school (days/week) | 0 (0; 3) | 0 (0;2) | 5 (4; 5) | 5 (2;5) | 0 (0; 5) | 0 (0;5) | 1 (0; 5) | 0 (0;4) | 3 (0; 5) | 3 (0;5) | 2 (0; 5) | 2 (0;5) | 5 (5;5) | 5 (3;5) |
| Walking to school (min/wk) | 0 (0; 30) | 0 (0;26) | 30 (30;80) | 30 (24;30) | 0 (0; 80) | 0 (0;55) | 6 (0; 30) | 0 (0;30) | 30 (0; 80) | 30 (0;80) | 30 (0; 80) | 30 (0;78) | 64 (30; 80) | 30 (30;80) |
| Sport participation (min/week) | 180 (60; 300) | 180 (120;300) | 120 ( 0; 240) | 180 (60;300) | 210 (120; 360) | 270 (150;413) | 225 (82.5; 330) | 180 (120;300) | 240 (120; 345) | 270 (180;390) | 270 (120; 360) | 270 (120;390) | 135 (0; 300) | 180 (90;300) |
| **Sedentary behaviors** | |  |  |  |  |  |  |  |  |  |  |  |  |  |
| Screen time FQ (min/day) | 176 (116; 257) | 15 (0; 60) | 193 (124; 257) | 180 (129; 240) | 223 (144;294) | 189 (124; 257) | 204 (129; 329) | 154 (99; 227) | 165 (124;227) | 159 (107; 219) | 193 (129; 274) | 159 (99; 234) | 159 (107; 249) | 150 (107; 223) |
| Screen time -24h recall (min/day) | 90 (60; 158) | 171 (111; 241) | 120 (60; 210) | 120 (60; 180) | 150 (90; 240) | 90 (60; 180) | 120 (60; 240) | 90 (30; 150) | 90 (60; 150) | 90 (60; 150) | 120 (60; 180) | 90 (30; 120) | 90 (60; 180) | 90 (30; 120) |
| TV time FQ(min/day) | 99 (60; 154) | 99 (69; 154) | 124 (77; 173) | 116 (77; 154) | 129 (77; 175) | 105 (69; 150) | 120 (69; 171) | 86 (47; 133) | 90 (69; 174) | 86 (60; 129) | 111 (69; 174) | 99 (60; 141) | 99 (60; 154) | 94 (64; 137) |
| TV time 24h recall (min/day) | 60 (30; 90) | 60 (30; 120) | 90 (60; 120) | 60 (60; 120) | 90 (60; 120) | 60 (30; 120) | 75 (30; 120) | 60 (30; 90) | 60 (30; 90) | 60 (30; 90) | 60 (30; 120) | 60 (30; 90) | 60 (30; 90) | 60 (30; 90) |
| Computer time FQ (min/day) | 71 (32; 110) | 60 (30; 99) | 60 (30; 99) | 69 (30; 99) | 88 (51; 137) | 77 (39; 120) | 99 (39; 159) | 60 (30; 116) | 69 (39; 101) | 69 (39; 99) | 69 (39; 120) | 51 (30; 99) | 69 (39; 111) | 56 (34; 94) |
| Computer time- 24h recall (min/day) | 0 (0; 60) | 15 (0; 60) | 30 (0; 60) | 30 (0; 60) | 30 (0; 90) | 30 (0; 90) | 60 (0; 120) | 30 (0; 60) | 30 (0; 60) | 30 (0; 60) | 30 (0; 60) | 0 (0; 60) | 30 (0; 60) | 30 (0; 60) |
| Sleeping habits(hr/night) | 9.7 (9.1;10.0) | 9.7 (9.3;10.0) | 8.6 (8.3;9.3) | 8.6 (8.3;9.3) | 9.3 (8.6;9.3) | 9.0 (8.6;9.3) | 9.7 (8.7;10.0) | 9.7 (9.3;10.0) | 9.3 (9.0;9.6) | 9.3 (9.0;;9.6) | 9.3 (8.7;9.6) | 9.0 (8.7;9.6) | 9.3 (9.0;10.0) | 9.3 (9.0;10.0) |
